# RelA/p65 inhibition prevents tendon adhesion by modulating inflammation, cell proliferation, and apoptosis

**DOI:** 10.1038/cddis.2017.135

**Published:** 2017-03-30

**Authors:** Shuai Chen, Shichao Jiang, Wei Zheng, Bing Tu, Shen Liu, Hongjiang Ruan, Cunyi Fan

**Affiliations:** 1 Department of Orthopaedics, Shanghai Jiao Tong University Affiliated Sixth People's Hospital, 600 Yishan Road, Shanghai 200233, People's Republic of China; 2 Department of Orthopaedics, Shandong Provincial Hospital Affiliated to Shandong University, No. 324 Jingwu Road, Jinan 250021, Shandong, People's Republic of China

## Abstract

Peritendinous tissue fibrosis which leads to poor tendon function is a worldwide clinical problem; however, its mechanism remains unclear. Transcription factor RelA/p65, an important subunit in the NF-*κ*B complex, is known to have a critical role in many fibrotic diseases. Here, we show that RelA/p65 functions as a core fibrogenic regulator in tendon adhesion and that its inhibition exerts an anti-fibrogenic effect on peritendinous adhesion. We detected the upregulation of the NF-*κ*B pathway in human tendon adhesion using a gene chip microarray assay and revealed the overexpression of p65 and extracellular matrix (ECM) proteins Collagen I, Collagen III, and *α*-smooth muscle actin (*α*-SMA) in human fibrotic tissues by immunohistochemistry and western blotting. We also found that in a rat model of tendon injury, p65 expression correlated with tendon adhesion, whereas its inhibition by small interfering (si)RNA prevented fibrous tissue formation and inflammatory reaction as evidenced by macroscopic, biomechanical, histological, immunohistochemical, and western blotting analyses. Furthermore, in cultured fibroblasts, p65-siRNA, p65-specific inhibitor, Helenalin and JSH23 suppressed cell proliferation and promoted apoptosis, whereas inhibiting the mRNA and protein expression of ECM components and cyclo-oxygenase-2, an inflammatory factor involved in tendon adhesion. Our findings indicate that p65 has a critical role in peritendinous tissue fibrosis and suggest that p65 knockdown may be a promising therapeutic approach to prevent tendon adhesion.

Flexor tendon adhesion, which is the generation of fibrotic tissue between the tendon and the surrounding synovial sheath after injury, is a complicated clinical problem.^1^ Tendon adhesion may compromise tendon gliding,^2^ causing unsatisfactory outcomes in up to 30–40% of flexor tendon injuries.^3^ Although a number of concepts regarding flexor tendon healing have been suggested,^4^ the mechanism of peritendinous tissue fibrosis has not been well defined.^2^ Therefore, despite numerous existing strategies to prevent fibrogenesis, an effective approach for the inhibition of tendon adhesion at the key points is still under development.^2, 5, 6, 7^

Fibrotic disease is an important but underestimated healthcare issue of great medical and socioeconomic impact.^8^ Fibrosis is manifested by excessive accumulation of extracellular matrix (ECM) components such as collagen and alpha smooth muscle actin (*α*-SMA) around injured tissue, which can lead to lasting scarring, pain, and dysfunction.^2, 8^ Persistent inflammation is known to activate fibrogenesis and is considered a major trigger in many fibrotic diseases.^8, 9, 10^ Nuclear factor (NF)-*κ*B, a key regulator of inflammation and cell survival,^11, 12^ has been reported to promote fibrogenesis in the liver and kidney.^13, 14, 15^ Among the five subunits of the NF-*κ*B complex, RelA/p65 is regarded as the crucial member of the canonical NF-*κ*B pathway.^16^ The role of p65 in fibrosis has been studied in many diseases. It has been reported that RelA/p65 is required for fibrogenesis in chronic pancreatitis of mice,^17^ whereas the inhibition of RelA significantly reduces liver fibrosis in a mouse model.^14^ These effects are likely mediated by RelA/p65 via regulation of cell proliferation, apoptosis, and inflammation critically involved in liver fibrosis.^13, 18, 19, 20^ In addition, RelA/p65 activation promoted cardiac fibrosis, inflammation, and apoptosis in heart failure.^21^ These findings may be relevant to fibrosis in other tissues as the core pathways in many fibrotic diseases are conserved,^10, 22^ suggesting that RelA/p65 may be an integral factor to tendon adhesion. Injured tendon heals following the classic wound healing process including inflammation, cell proliferation, and apoptosis.^4^ Therefore, p65 as a key signaling molecule of the NF-*κ*B pathway may modulate the tendon repair process and, more importantly, fibrosis of peritendinous tissue. However, the role of RelA/p65 in tendon adhesion has not been described.

The aim of this study was to determine whether RelA/p65 is essential for tendon adhesion progression. The correlation between p65 and tendon adhesion was analyzed in human samples and confirmed in a rat model of tendon injury. The mechanism underlying p65 role in peritendinous fibrosis was evaluated *in vivo* and *in vitro* by manipulating p65 activity using small interfering (si)RNA and pharmacological inhibitors. Our results suggest that RelA/p65 promotes tendon adhesion by inducing fibroblast growth, expression of ECM components, and pro-inflammatory mediators.

## Results

### NF-*κ*B signaling pathway is involved in tendon adhesion in humans

**Figure 1:**
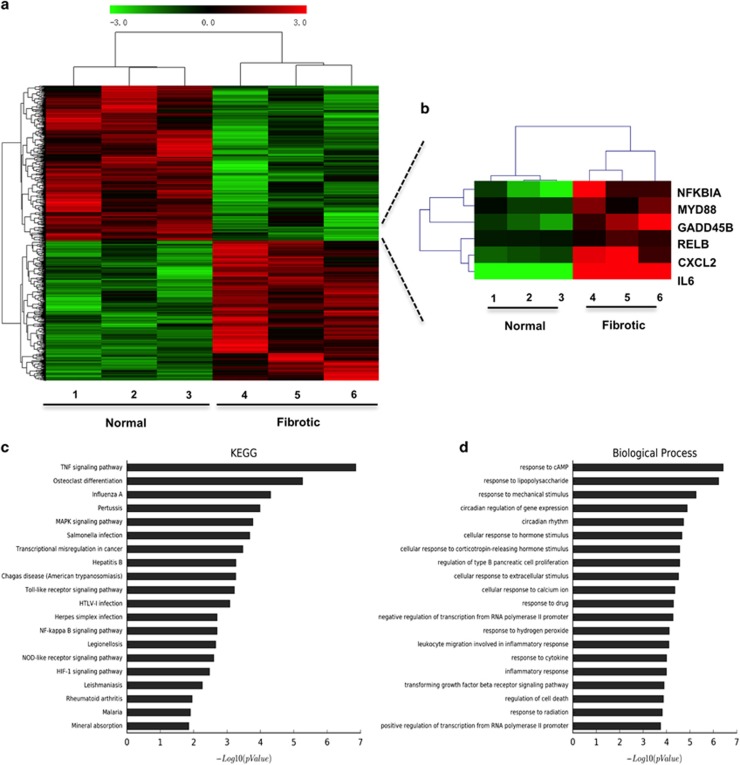
Gene expression profile of tendon fibrotic tissues. (**a**) results of gene chip microarray in normal and fibrotic samples from patients. (**b**) Heat map depicting the gene expression profiles of NF-*κ*B pathway in normal and fibrotic samples. Red, high expression; black, intermediate expression; green, lower expression. (**c**) The top 20 canonical pathways enriched in the upregulated genes by KEGG pathway analysis. (**d**) The top 20 biological process enriched in the upregulated genes by GO analysis of upregulated genes

To determine the mechanism of peritendinous fibrosis, gene expression was analyzed in fibrotic tissues collected from patients who underwent tendon release using gene chip microarray; normal tissue from the same patient was used as control (Figure 1a). A heat map depicting gene expression in normal and fibrotic tissues indicated that transcripts of six genes, *NFKBIA*, *MYD88*, *GADD45B*, *RELB*, *CXCL2*, and *IL6* participating in NF-*κ*B signaling were significantly increased in fibrotic tissues (Figure 1b), which was confirmed by real-time PCR (Supplementary Figure 1). We further used Kyoto Encyclopedia of Genes and Genomes (KEGG) pathway and gene ontology (GO) analyses to determine the roles of upregulated genes in pathways and molecular functions. NF-*κ*B pathway was enriched in KEGG pathway analysis of upregulated genes (Figure 1c). In addition, inflammatory response and regulation of cell death were both enriched in the biological process analysis of upregulated genes (Figure 1d). The genes related to the NF-*κ*B pathway showed significant difference in expression between the two tissue types (Supplementary Figure 1). These findings suggest that the NF-*κ*B pathway is closely involved in fibrogenesis around the tendon, which is consistent with previous reports about the important role of NF-*κ*B in fibrosis.^13, 14, 15^

### Human fibrotic tissues exhibited increased p65 expression in tendon adhesion

**Figure 2:**
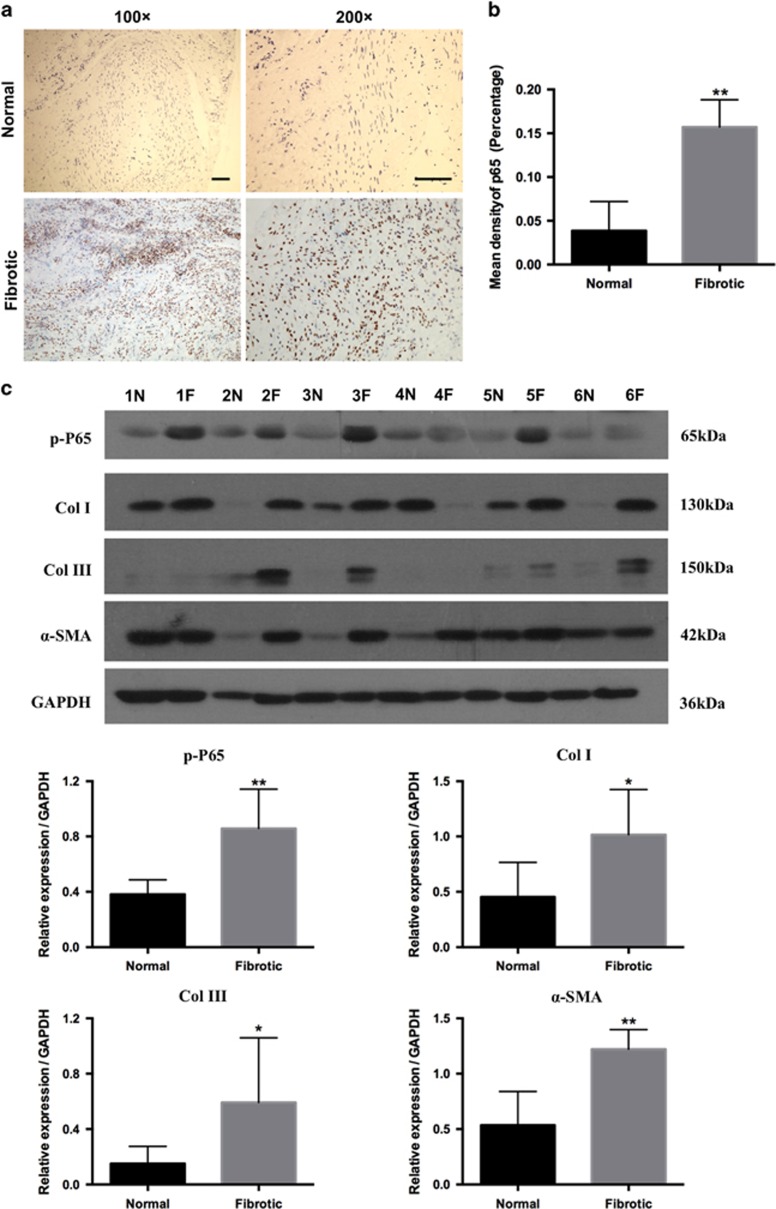
The expression of RelA/p65 in human samples. (**a**) The expression of p65 was detected by immunohistochemistry in human samples. Bars indicate 200 *μ*m. (**b**) Significant difference in p65 expression level between normal and fibrotic tissues. Results were expressed as percentage density for five independent samples, and *P-*values were determined using paired *t*-test. **P<0.01. (**c**) Western blot analysis of the relative levels of p-p65, Col I, Col III, and *α*-SMA protein expression in normal (N) and fibrotic (F) tissues of six patients. Densitometric analysis of p-p65, Col I, Col III, and *α*-SMA expression. Statistical significance was calculated using paired Student *t*-test for ten pairs of samples. The data are shown as the means±S.D. *P<0.05. **P<0.01

Immunohistochemistry analysis revealed that the expression of p65 in fibrotic samples (Figure 2a) was significantly increased compared with normal tissues (*P*<0.01, Figure 2b). Western blotting analysis showed that the levels of phosphorylated (activated) form of p65 (p-p65) and ECM components Collagen I (COL I), Collagen III (COL III), and *α*-smooth muscle actin (*α*-SMA) were increased in fibrotic samples and reached statistical significance (Figure 2c). These data suggest that in tendon adhesion, p65 activation may correspond to the upregulation of ECM markers indicating fibrogenesis, which is consistent with an important role of p65 in fibrosis.^14, 17^ Thus, p65 may modulate the development of tendon adhesion.

### RelA/p65 is related to peritendinous fibrosis in rats

**Figure 3:**
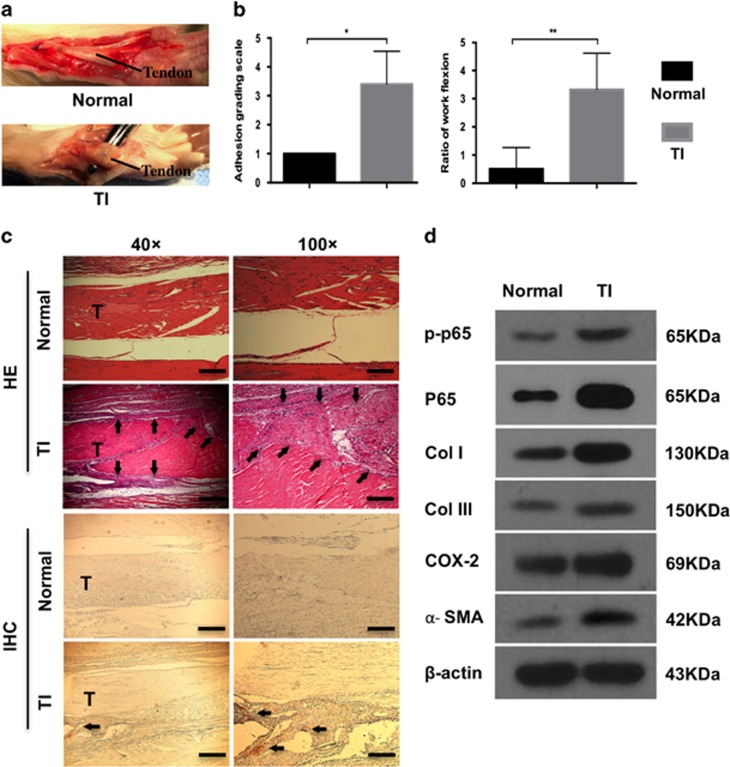
The increased p65 expression in rat tendon adhesion. (**a**) Macroscopic images of tendons in normal and TI groups. (**b**) The adhesion grading score and ratio of work flexion for adhesion evaluation. The data are shown as the means±S.D. Statistical significance was calculated using paired Student *t*-test for five independent samples. *P<0.05. **P<0.01. (**c**) Histology of adhesion and immunohistochemistry of p65. T, tendon. Arrows indicate tendon adhesion in HE and p65 overexpression in IHC. × 40, bars indicates 500 *μ*m. × 100, bars indicates 200 *μ*m. (**d**) Western blot analysis of the levels of p-p65, p65, Col I, Col III, COX-2 and *α*-SMA expression

We further confirmed the findings of human tissues in a rat model of tendon adhesion. Tendon injury was inflicted in rats and examined three weeks post-operation; normal tendons served as control. Dense fibrotic tissue requiring sharp dissection for separation from the tendon was observed in the tendon injury (TI) group, whereas smooth tendon surface was found in the normal group (Figure 3a). Gross observation of peritendinous adhesion revealed significant difference between the TI and normal control group (*P*<0.01; Figure 3b). In addition, the work of flexion indicating the severity of tendon adhesion was significantly higher in the TI group compared to the normal group (Figure 3b). Histological evaluation showed that in the TI group, severe adhesion formed between the tendon and surrounding tissue accompanied by massive infiltration of inflammatory cells and dense fibrosis, whereas in the normal group, a clear gap was observed between the tendon and surrounding tissue (Figure 3c). Furthermore, enhanced expression of p65 in fibrotic tissue was detected in the TI group by immunohistochemistry (Figure 3c). Taken together, these data indicated that tendon injury led to tissue adhesion around the tendon, which correlated with p65 expression. To further confirm this connection, the expression of p-p65, p65, COL I, COL III, *α*-SMA, and cyclo-oxygenase-2 (COX-2) was evaluated by western blotting (Figure 3d). The expression of ECM components was enhanced in the TI group compared with the normal group, indicating fibrogenesis. Furthermore, COX-2, an inflammatory factor in tendon adhesion,^2^ was also increased. Importantly, higher expression of p-p65 and p65 in the TI group compared to the normal group was observed, indicating the involvement of RelA/p65 in the formation of fibrotic tissue. Overall, these findings suggest that p65 is related to inflammation and ECM deposition in tendon adhesion.

### P65 inhibition prevents tendon adhesion in rats

**Figure 4:**
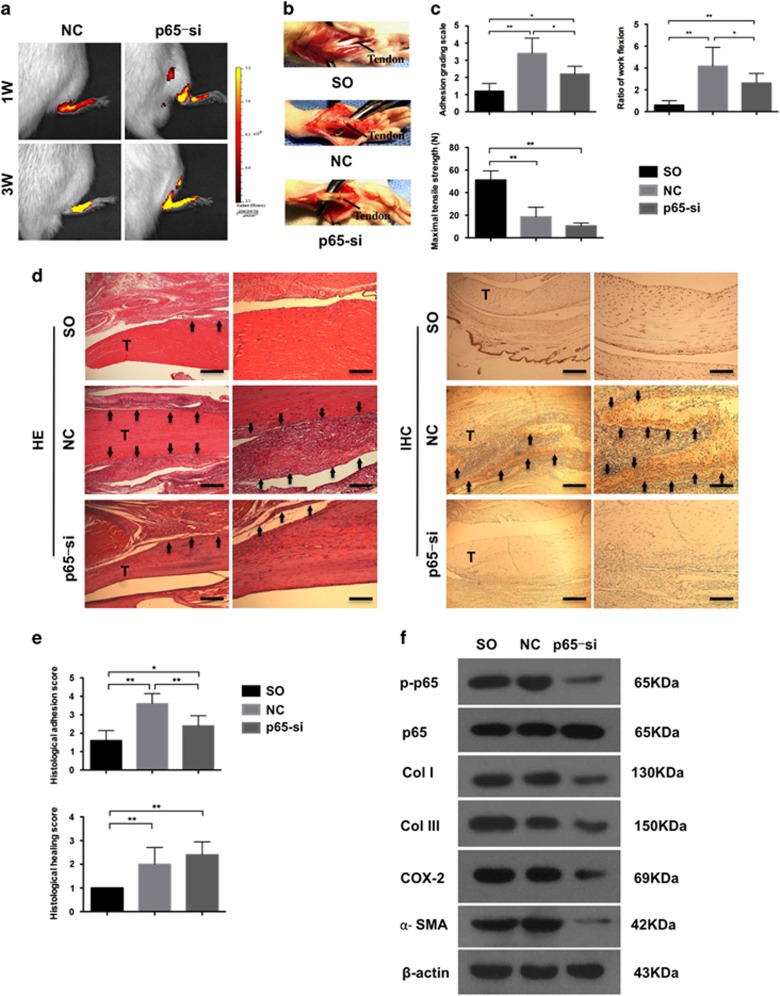
P65 knockdown by siRNA in rat tendon adhesion. (**a**) Fluorescent images showing the location and activity of siRNA. (**b**) Macroscopic images showing adhesion in SO group, NC group, and p65-siRNA group (p65-si). (**c**) The adhesion grading score, ratio of work flexion and maximal tensile strength. The data are shown as the means±S.D. Statistical significance was calculated using one-way ANOVA for five independent samples. *P<0.05. **P<0.01. (**d**) Histology of adhesion and immunohistochemistry of p65. T, tendon. Arrows indicate tendon adhesion in HE and p65 overexpression in IHC. × 40, bars indicates 500 *μ*m. × 100, bars indicates 200 *μ*m. (**e**) Histological adhesion score and histological healing score. The data are shown as the means±S.D. Statistical significance was calculated using one-way ANOVA for five independent samples. (**f**) Western blot analysis of the levels of p-p65, p65, Col I, Col III, COX-2 and *α*-SMA expression

To validate the involvement of p65 in tendon adhesion, p65 inhibition was performed in the feet of rats by subcutaneous injection of p65-siRNA selected by real-time PCR and western blotting (Supplementary Figure 2B). Rats were randomly assigned to three groups: sham-operated (SO) group, negative control (NC) group, and p65-siRNA (p65-si) group. One and three weeks after surgery, the localization and activity of siRNA were confirmed by fluorescence imaging (Figure 4a). Smooth tendon surface was observed in SO group with little fibrotic tissue. However, thick fibrotic tissues which could be distinguished from the tendon only by sharp dissection were found around the tendon in NC group (Figure 4b). Although there was only few fibrotic tissue which required only blunt dissection in the p65-si group (Figure 4b). Moreover, the adhesion grading score in the SO group was significantly lower compared with the NC group (*P*<0.01) and p65-si group (*P*<0.05); however, the fibrotic score was significantly decreased in the p65-si group compared with the NC group (*P*<0.05) (Figure 4c). Consistent with these results, the work of flexion also showed decreased fibrogenesis in the p65-si group (*P*<0.05, Figure 4c). Overall, these findings indicate that p65 knockdown was effective in alleviating tendon adhesion, disclosing an important role of p65 in the development of peritendinous fibrosis *in vivo*. It should be mentioned that no significant difference was observed between NC and p65-si rats regarding the maximal tensile strength (Figure 4c), indicating that p65 inhibition did not influence tendon healing.

Histology findings showed that rats treated with control siRNA (NC) had more fibrosis and infiltrated inflammatory cells than the other groups (Figure 4d). Significant difference was also observed in the histological adhesion score, indicating increased fibrogenesis in the NC group compared to the other two groups (Figure 4e). At the same time, there was no difference in tendon healing according to histological tendon healing score between the NC and p65-si groups (Figure 4e), which was consistent with the biomechanical test results. These findings show that p65-siRNA inhibits fibrogenesis and relieves inflammation without affecting tendon healing. Furthermore, the expression of p65 determined by immunohistochemistry was higher in the NC group than in the other two groups (Figure 4d). However, the treatment with p65-siRNA decreased p65 expression and fibrous tissue formation, supporting the role of p65 in fibrosis and suggesting the effectiveness of targeting p65 as a therapeutic strategy for tendon adhesion.

Next, the expression of p-p65, p65, COL I, COL III, *α*-SMA, and COX-2 in peritendinous tissue was examined by western blotting (Figure 4f). Fibrotic tissues could be collected in the NC and p65-siRNA groups; however, in the untreated SO group, almost no fibrotic tissue formation was observed (Figure 4a), and it was not possible to harvest peritendinous fibrotic tissue for evaluation. At the same time, subcutaneous fibrous tissues formed in the process of skin incision repair was found during dissection in SO rats. Thus, the SO group represented control for natural would healing process, to compare tendon adhesion formation and skin wound healing as it has been reported that injured tendon heals following the classic wound healing process. Because the formation of peritendinous and subcutaneous tissues in the NC and SO groups, respectively, showed a similar trend (Figure 4f), we may infer that tendon injury repairs following a wound healing process.^4^ As there was scarcely any fibrogenesis around the tendon in the SO group (Figure 4a), it may be tentatively inferred that the expression of COL I, COL III, COX-2, and *α*-SMA in the SO group was reduced compared to the NC group. We speculate that the direct tendon injury mainly induces adhesion formation, as hardly no adhesion was found in the SO group compared to the NC group. More importantly, p65-si reduced the levels of p65 and ECM components COL I, COL III, and *α*-SMA as well as of COX-2, indicating that targeting p65 decreases fibrosis and inflammation.

### P65-siRNA influences fibroblasts proliferation, apoptosis, and inflammation *in vitro*

**Figure 5:**
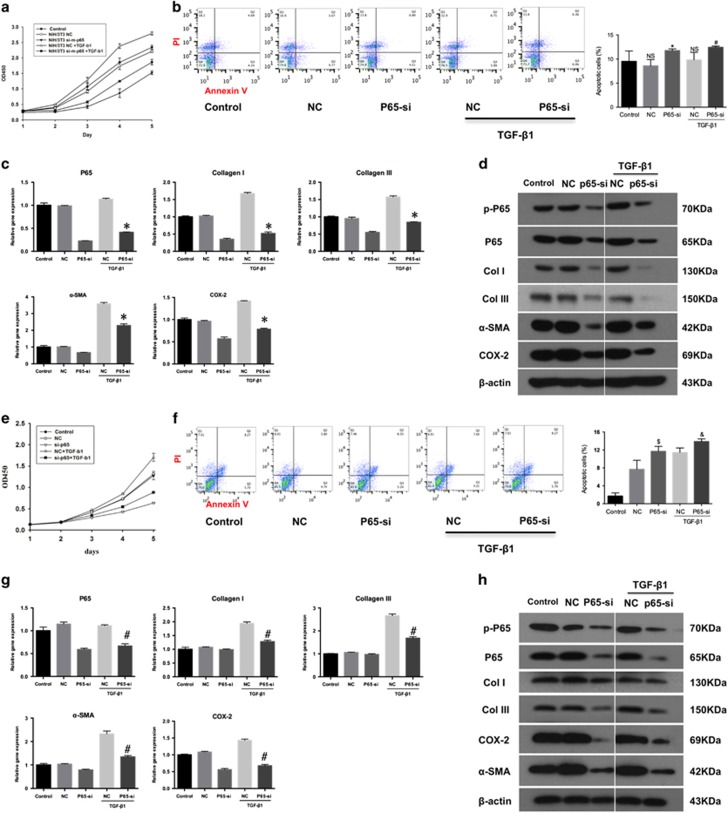
P65 inhibition by siRNA influences cell proliferation, apoptosis, and inflammation *in vitro*. (**a**) Fbs proliferation. (**b**) The apoptosis of Fbs by flow cytometry. The data are shown as the means±S.D. Statistical significance was calculated by one-way ANOVA for five independent samples. NS, no significance compared control group. *P<0.05 compared with NC group, ^#^*P*<0.05 compared with NC+TGF-*β*1 group. (**c**) The RT-PCR analysis of the mRNA levels of p65, Col I, Col III, COX-2 and *α*-SMA expression in Fbs. The data are shown as the means±S.D. Statistical significance was calculated by one-way ANOVA for five independent samples. *P<0.05 compared with NC+TGF-*β*1 group. (**d**) Western blot analysis of the protein levels of p-p65, p65, Col I, Col III, COX-2 and *α*-SMA expression in Fbs. (**e**) Tcs cells (Tcs) proliferation. (**f**) The apoptosis of Fbs by flow cytometry. The data are shown as the means±S.D. Statistical significance was calculated using one-way ANOVA for five independent samples. ^$^*P*<0.01 compared with NC group, ^&^*P*<0.05 compared with NC+TGF-*β*1 group. (**g**) The RT-PCR analysis of the mRNA levels of p65, Col I, Col III, COX-2 and *α*-SMA expression in Tcs. The data are shown as the means±S.D. Statistical significance was calculated by one-way ANOVA for five independent samples. ^#^*P*<0.05 compared with NC+TGF-*β*1 group. (**h**) Western blot analysis of the levels of p-p65, p65, Col I, Col III, COX-2 and *α*-SMA expression in Tcs

To address the mechanisms underlying p65 activity in tendon adhesion development, NIH3T3 fibroblasts (Fbs) and tendon cells (Tcs) were transfected with p65-siRNA selected for p65 inhibition by real-time PCR and western blotting (Supplementary Figure 2B). Transforming growth factor beta 1 (TGF-*β*1) known for its role in fibrogenesis was used to imitate the *in vivo* microenvironment during fibrosis.^23^ Cells were divided into five groups: control, NC, p65-si, NC+TGF-*β*1, and p65-si+TGF-*β*1. Transfection with p65-si significantly reduced cell proliferation (Figure 5a) and increased apoptosis (Figure 5b) in Fbs cultures irrespectively of TGF-*β*1 pre-treatment, suggesting that p65 supported Fbs growth during fibrogenesis through inhibition of apoptosis. Furthermore, the reduction in p65 mRNA in p65-si+TGF-*β*1 group compared to NC+TGF-*β*1 group (0.37-fold, *P*<0.01; Figure 5c) corresponded to that in mRNA expression of COL I, COL III, *α*-SMA, and COX-2 (0.31-, 0.54-, 0.64, and 0.55-fold, respectively; *P*<0.01 for all, Figure 5c). Consistently, western blotting analysis showed that the expression of p-p65, p65, ECM markers COL I, COL III, and *α*-SMA, and pro-inflammatory COX-2 was decreased after the treatment with p65-siRNA (Figure 5d), indicating the suppression of fibrogenesis and inflammation, which is in agreement with the *in vivo* findings. These results indicate that the siRNA targeting RelA/p65 suppressed fibrogenic and pro-inflammatory activity of Fbs. As fibroblasts have the main role in fibrosis,^2^ these findings suggest that the p65 subunit of the NF-*κ*B complex may modulate tendon adhesion by regulating fibroblast proliferation, apoptosis, and inflammation.

Similar results were obtained in Tcs. P65-siRNA inhibited Tcs proliferation (Figure 5e) and increased apoptosis (Figure 5f). In addition, p65-siRNA significantly decreased p65 mRNA expression in p65-si+TGF-*β*1 group comparing with NC+TGF-*β*1 group (0.60-fold, *P*<0.01; Figure 5g), which corresponded to the sifnificant reduction in mRNA expression COL I, COL III, *α*-SMA, and COX-2 (0.66-, 0.63-, 0.58-, and 0.47-fold, respectively; *P*<0.01 for all, Figure 5g). Furthermore, p65-siRNA downregulated the protein expression of p-p65 and p65, ECM markers, and COX-2 (Figure 5h) with TGF-*β*1 pre-treatment. These results indicated that p65-siRNA inhibited proliferation, promoted apoptosis, and decreased ECM production in Tcs, which may be unfavorable for tendon healing.

### P65 inhibitor Helenalin modulates fibroblasts proliferation, apoptosis, and inflammation *in vitro*

**Figure 6:**
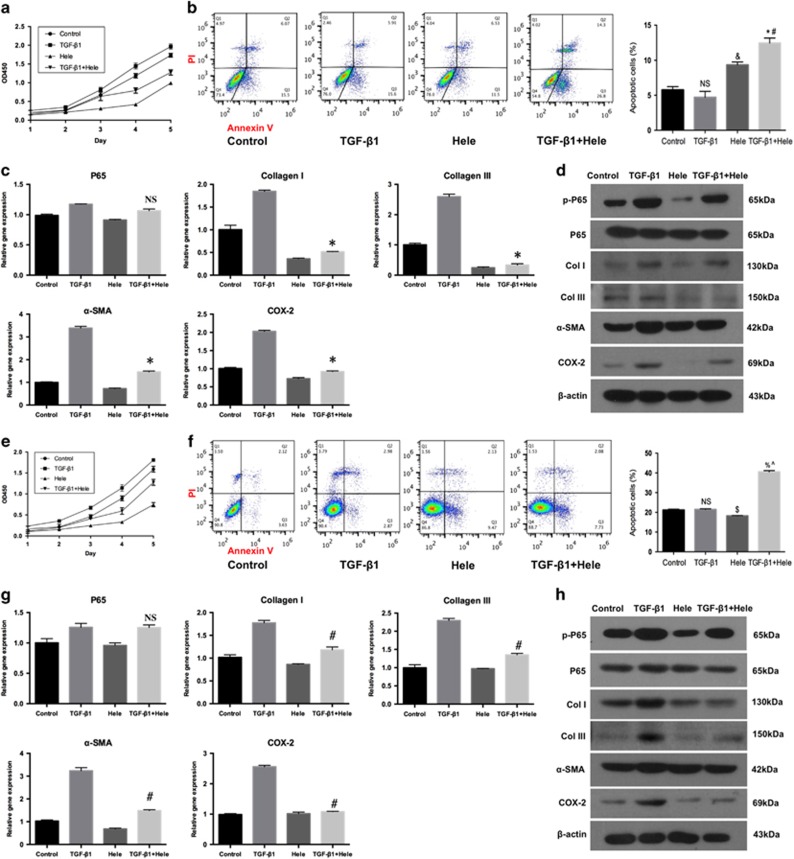
P65 inhibitor Helenalin modulates cell proliferation, apoptosis, and inflammation *in vitro*. (**a**) Fbs proliferation. (**b**) The apoptosis of Fbs by flow cytometry. The data are shown as the means±S.D. Statistical significance was calculated using one-way ANOVA for five independent samples. NS, no significance compared with control. ^&^*P*<0.01 compared with control, *P<0.01 compared with control, ^#^*P*<0.01 compared with TGF-*β*1 group. (**c**) The RT-PCR analysis of the mRNA levels of p65, Col I, Col III, COX-2 and *α*-SMA expression in Fbs. The data are shown as the means±S.D. Statistical significance was calculated by one-way ANOVA for five independent samples. NS, no significance compared with TGF-*β*1 group. *P<0.05 compared with TGF-*β*1 group. (**d**) Western blot analysis of the protein levels of p-p65, p65, Col I, Col III, COX-2 and *α*-SMA expression in Fbs. (**e**) Tcs proliferation. (**f**) The apoptosis of Tcs by flow cytometry. The data are shown as the means±S.D. Statistical significance was calculated using one-way ANOVA for five independent samples. NS, no significance compared with control. ^$^*P*<0.01 compared with control, ^%^*P*<0.01 compared with control group, ^^^*P*<0.01 compared with TGF-*β*1 group. (**g**) The RT-PCR analysis of the mRNA levels of p65, Col I, Col III, COX-2 and *α*-SMA expression in Tcs. The data are shown as the means±S.D. Statistical significance was calculated by one-way ANOVA for five independent samples. NS, no significance compared with TGF-*β*1 group. ^#^*P*<0.05 compared with TGF-*β*1 group. (**h**) Western blot analysis of the levels of p-p65, p65, Col I, Col III, COX-2 and *α*-SMA expression in Tcs

To further investigate the role of p65 in tendon adhesion, Fbs and Tcs cultures pre-treated with TGF-*β*1or not were incubated with Helenalin, a specific inhibitor of p65. Although TGF-*β*1 promoted the proliferation of Fbs, Helenalin markedly reduced cell proliferation in both TGF-*β*1-treated and -untreated cultures (Figure 6a). Flow cytometry analysis of apoptosis did not detect any difference between cultures treated or not with TGF-*β*1, whereas Helenalin significantly increased apoptosis irrespectively of TGF-*β*1 presence compared to control (*P*<0.01, Figure 6b). Interestingly, although TGF-*β*1 did not influence the rate of apoptosis in Fbs, it potentiated the effect of Helenalin as evidenced by the upregulation of apoptosis in cells treated with TGF-*β*1+ Helenalin compared to those treated with Helenalin alone (*P*<0.01; Figure 6b). These results indicated that the inhibition of p65 promoted Fbs apoptosis, especially in the pro-fibrogenic environment. Furthermore, Helenalin did not reduced the expression of p65 mRNA in TGF-*β*1+Helenalin group compared to the TGF-*β*1 group (Figure 6c), which is consistently with previous report that Helenalin selectively alkylates the p65 subunit.^24^ Meanwhile, mRNA expression of COL I, COL III, *α*-SMA, and COX-2 in the TGF-*β*1+Helenalin group was significantly decreased compared to the TGF-*β*1 group (0.20-, 0.10-, 0.21-, and 0.36-fold, respectively; *P*<0.01 for all, Figure 6c). In addition, Helenalin markedly decreased the protein expression of p-p65, ECM components COL I, COL III, *α*-SMA, and COX-2 in Fbs treated or not with TGF-*β*1 (Figure 6d). Overall, these results confirm that p65 controls tendon adhesion by regulating Fbs proliferation, apoptosis, and inflammation.

To examine whether Helenalin could potentially affect tendon healing, the same experiments were performed in Tcs. Similar to Fbs, Helenalin reduced Tcs proliferation (Figure 6e) and enhanced apoptosis of Tcs stimulated with TGF-*β*1 than TGF-*β*1 group (Figure 6f). However, Helenalin significantly inhibited apoptosis in unstimulated Tcs (Figure 6f), which was in contrast with the findings in p65-siRNA-transfected cells (Figure 5f), suggesting the existence of other mechanisms regulating Tcs apoptosis. In addition, Helenalin did not affect the p65 mRNA expression of Tcs in TGF-*β*1+Helenalin group compared with TGF-*β*1 group (Figure 6g). However, the mRNA expression of COL I, COL III, *α*-SMA, and COX-2 of TGF-*β*1+Helenalin group was significantly decreased than TGF-*β*1 group (0.67-, 0.59-, 0.46-, and 0.42-fold, respectively; *P*<0.01 for all; Figure 6g). Similar to p65-siRNA, Helenalin decreased the expression of p-p65, COL I, COL III, *α*-SMA, and COX-2; however, no difference was observed in expression, which might be the reason that *α*-SMA is a specific marker for fibroblasts, not tendon cells.

### P65 inhibitor JSH23 regulates fibroblasts proliferation, apoptosis, and inflammation *in vitro*

**Figure 7:**
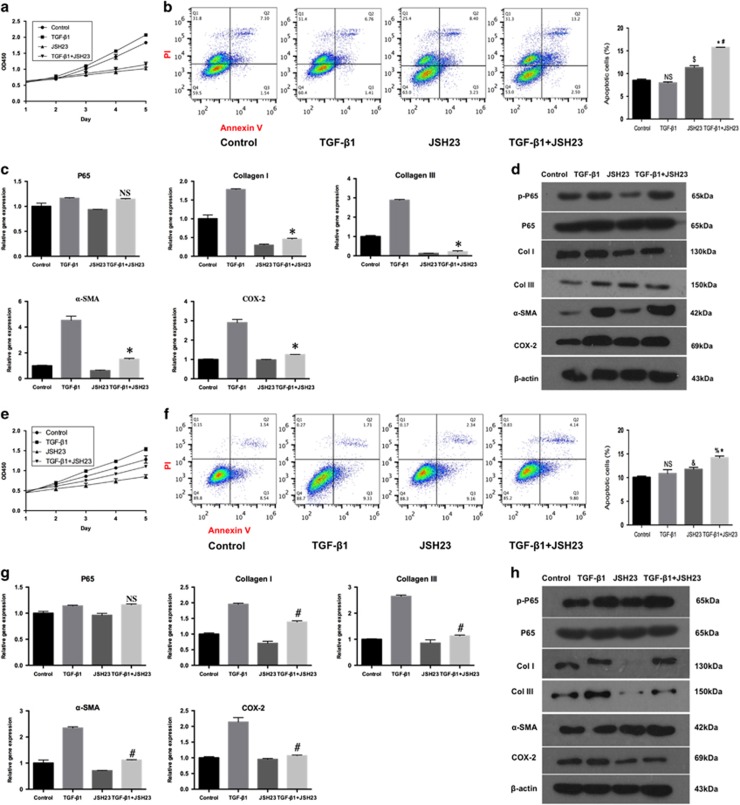
P65 inhibitor JSH23 regulates cell proliferation, apoptosis, and inflammation *in vitro*. (**a**) Fbs proliferation. (**b**) The apoptosis of Fbs by flow cytometry. The data are shown as the means±S.D. Statistical significance was calculated using one-way ANOVA for five independent samples. NS, no significance compared with control. ^$^*P*<0.01 compared with control, *P<0.01 compared with control, ^#^*P*<0.01 compared with TGF-*β*1 group. (**c**) The RT-PCR analysis of the mRNA levels of p65, Col I, Col III, COX-2 and *α*-SMA expression in Fbs. The data are shown as the means±S.D. Statistical significance was calculated by one-way ANOVA for five independent samples. NS, no significance compared with TGF-*β*1 group. *P<0.05 compared with TGF-*β*1 group. (**d**) Western blot analysis of the protein levels of p-p65, p65, Col I, Col III, COX-2 and *α*-SMA expression in Fbs. (**e**) Tcs proliferation. (**f**) The apoptosis of Tcs by flow cytometry. The data are shown as the means±S.D. Statistical significance was calculated using one-way ANOVA for five independent samples. NS, no significance compared with control. ^&^*P*<0.01 compared with control, ^%^*P*<0.01 compared with control group, *P<0.01 compared with TGF-*β*1 group. (**g**) The RT-PCR analysis of the mRNA levels of p65, Col I, Col III, COX-2 and *α*-SMA expression in Tcs. The data are shown as the means±S.D. Statistical significance was calculated by one-way ANOVA for five independent samples. NS, no significance compared with TGF-*β*1 group. ^#^*P*<0.05 compared with TGF-*β*1 group. (**h**) Western blot analysis of the levels of p-p65, p65, Col I, Col III, COX-2 and *α*-SMA expression in Tcs

To further evaluate the role of p65 in peritendinous fibrosis, Fbs and Tcs pre-treated or not with TGF-*β*1 were cultured with JSH23 (another specific p65 inhibitor). JSH23 reduced Fbs proliferation irrespective of TGF-*β*1 treatment (Figure 7a). Although there was no difference in apoptosis between TGF-*β*1-treated and untreated cells (Figure 7b), JSH23 induced apoptosis in both Fb groups compared to control (*P*<0.01, Figure 7b), and TGF-*β*1 potentiated the apoptotic effect of JSH23 (*P*<0.01; Figure 7b). These findings also confirmed that p65 inhibition promoted Fbs apoptosis, particularly in the pro-fibrogenic condition. In addition, JSH23 treatment did not affect the expression of p65 mRNA (Figure 7c) as it inhibits p65 by preventing its nuclear translocation.^25^ However, the mRNA expression levels of COL I, COL III, *α*-SMA, and COX-2 in the TGF-*β*1+ JSH23 group were all significantly decreased compared with the TGF-*β*1 group (0.26-, 0.08-, 0.33-, and 0.43-fold, respectively; *P*<0.01 for all). Furthermore, JSH23 markedly decreased the protein expression of p-p65, ECM components COL I, COL III, and *α*-SMA, and COX-2 in Fbs treated or not with TGF-*β*1 (Figure 7d). These results indicated the inhibition of p65 by JSH23 suppressed the mRNA and protein expression of ECM components COL I, COL III, and *α*-SMA, and COX-2 in Fbs in the pro-fibrogenic environment. Overall, these findings confirm that p65 controls tendon adhesion by regulating Fbs proliferation, apoptosis, and inflammation.

To examine whether JSH23 could restrict tendon healing, the same experiments were carried out in Tcs. JSH23 also suppressed proliferation (Figure 7e) and increased apoptosis of Tcs stimulated with TGF-*β*1 (Figure 7f). similar to p65-siRNA, but contrary to Helenalin, JSH23 significantly promoted apoptosis in unstimulated Tcs (Figure 7f), which was opposite to cells treated by Helenalin. In addition, the mRNA expression levels of COL I, COL III, and *α*-SMA, and COX-2 in the TGF-*β*1+JSH23 group were significantly decreased compared with the TGF-*β*1 group (0.71-, 0.43-, 0.48-, and 0.50-fold, respectively; *P*<0.01 for all, Figure 7g), however, the expression of p65 mRNA expression was not decreased (Figure 7g). Futhermore, JSH23 also decreased the protein expression of p-p65, COL I, COL III, and COX-2 (Figure 7h).

## Discussion

Our work revealed, for the first time, the role of RelA/p65 in tendon adhesion. We showed that in human fibrotic tissues, the NF-*κ*B signaling pathway was activated and the expression of RelA/p65 induced. These results were confirmed in a rat model of tendon injury, where p65 expression was increased in peritendinous fibrotic tissue in parallel with the upregulation of ECM deposition and the level of pro-inflammatory factor COX-2. Importantly, fibrogenesis around rat tendons was efficiently inhibited by p65-specific siRNA, which corresponded to decreased expression of ECM and inflammatory markers, highlighting p65 the role in tendon adhesion. Finally, *in vitro* experiments suggested that p65 regulated the proliferation of fibroblasts through inhibition of apoptosis and induced the expression of ECM components and pro-inflammatory COX-2, suggesting a mechanism underlying p65 role in tendon adhesion.

Fibrosis is considered a wound healing reaction to injury, in which the balance between tissue repair and excessive ECM accumulation is changed towards the latter.^15^ Fibrogenesis is initiated by tissue injury, which triggers inflammation by recruiting pro-inflammatory factors.^15^ The NF-*κ*B pathway is a key regulator of inflammatory response as its activation induces the transcription of pro-inflammatory mediators. Our results indicate that the expression of signaling molecules involved in the NF-*κ*B pathway, including p65, is upregulated in fibrous tissue formed around the tendon both in human and rat samples, suggesting a link between NF-*κ*B activation and tendon adhesion. Importantly, we could successfully prevent peritendinous fibrosis in rats by p65-siRNA, which confirms the involvement of p65, suggesting it as a target in tendon adhesion treatment. Our results are consistent with previous findings that p65 was essential for fibrogenesis in mouse pancreatitis^17^ and that liver fibrosis in mice was reduced by p65 inhibition.^14^

We also addressed the mechanism underlying p65 activity in peritendinous fibrosis, and revealed that in fibroblasts, which are the main players in fibrogenesis, p65 promoted cell proliferation, inhibited apoptosis, and induced the expression of ECM and pro-inflammatory markers. Consistent with our findings, p65 was shown to promote liver fibrosis by regulating inflammation, proliferation, and apoptosis.^13, 14, 18, 19, 20^ Thus, p65 activation in fibroblasts may aggravate tendon adhesion by promoting cell growth and inducing inflammation.

In this study, we used three methods for fibroblasts p65 inhibition including p65-siRNA, Helenalin and JSH23. Thus, p65-siRNA targeted p65 by interfering with p65 gene expression via mRNA degradation after transcription, resulting in translation blockage, whereas Helenalin selectively alkylated the p65 subunit, and JSH23 prevented NF-*κ*B nuclear translocation. The difference in inhibitory mechanisms may account for some discrepancy in the results. However, our results clearly show that irrespective of the method used for p65 inhibition, the expression of p-p65, ECM markers COL I, COL III, and *α*-SMA, and pro-inflammatory COX-2 was decreased, indicating that targeting RelA/p65 suppressed fibrogenic and pro-inflammatory processes. Here we show that the activated p65 (p-p65) may regulate the gene and protein expression of the markers which are involved in tendon adhesion, such as COL I, COL III, *α*-SMA and COX-2. The downregulation of p-p65 may be caused by different reasons: decreased expression of total p65 by siRNA, suppression of p65 activity through alkylation by Helenalin, and reduction of p65 phosphorylation in the nuclei through decrease of p65 nuclear translocation by JSH23.

Although p65 inhibition by all three means decreased proliferation of tendocytes *in vitro*, p65-siRNA did not significantly influenced maximal tendon strength *in vivo*, indicating that p65 interference did not negatively affect tendon healing. One reasonable explanation for the observed discrepancy may be that the amount of injected p65-siRNA was sufficient to influence tendon cells activity, but insufficient to affect tendon healing. Besides, regulatory mechanisms other than p65-mediated signaling may be involved in tendon healing. Further studies are required to determine the effect of p65 inhibition on the function of tendon healing. Moreover, in our experiments, Helenalin reduced apoptosis of tenocytes but induced it in fibroblasts under normal conditions, which may be because of differential effects of p65 alkylation by Helenalin in these cell types. In addition, the response to Helenalin may differ in primary cells (tenocytes) and cell lines (fibroblasts cell line). Further studies are needed to test the potential mechanisms. Therefore, further studies are required to determine the effect of p65 inhibition on the function of tendon healing.

Currently, there is no effective treatment for tendon adhesion, and the development of a therapeutic approach to prevent peritendinous tissue fibrosis remains a challenge for clinical research. Physical barriers,^6, 26, 27^ chemical agents,^5, 28^ and their combination^29, 30^ have been applied with uneven results. However, these methods do not target the critical point. Our findings warrant further investigation of p65 inhibition as a strategy to prevent tendon adhesion. However, this study had limitations. First, the number of human samples used for gene chip assay was small, and second, the dose of p65-siRNA used for *in vivo* injections was not optimized and needs to be determined in further studies.

## Materials and methods

### Human samples

Fibrotic tissues were collected around the tendon from 30 patients who underwent tendon release 4–6 months after injury between February 2014 and November 2015. Control tissues were obtained from healthy areas of the peritendinous tissue in the same patient. The median age of the patients was 35.8 years (range, 24–47 years). This study was approved by the ethics committee of Shanghai Jiao Tong University Affiliated Sixth People's Hospital. The analysis was conducted following the principles of the Declaration of Helsinki. Informed consent was obtained from all patients.

### Gene chip microarray assay

Three pairs of human samples, including normal and fibrotic tissues, were shock-frozen in liquid nitrogen, pulverized, and dissolved in Trizol (Life Technologies, Carlsbad, CA, USA) for total RNA isolation. RNA concentration was quantified using the NanoDrop ND-2000 instrument (Thermo Fisher Scientific, Rockford, IL, USA), and RNA integrity was assessed using Agilent Bioanalyzer 2100 (Agilent Technologies, Santa Clara, CA, USA).

### Gene expression analysis

The Affymetrix GeneChip Command Console software version 4.0 (Affymetrix) was used to extract raw data. RNA normalization was performed using the Expression Console software version 1.3.1 (Affymetrix, Santa Clara, CA, USA). Basic analysis was performed using the Genesrping software version 13.1 (Agilent Technologies). Differentially expressed genes were then identified by fold change ⩾2 and a *P-*value ⩽0.05 calculated by *t*-test. KEGG pathway and GO analyses were applied to determine the roles of upregulated genes in pathways and molecular functions.

### Real-time quantitative RT-PCR

Total RNA was extracted with Trizol (Invitrogen, Carlsbad, CA, USA) and complementary (c)DNA was synthesized using the iScript cDNA Synthesis Kit (Bio-Rad, Hercules, CA, USA) and GeneAmp PCR System 9700 (Applied Biosystems, Carlsbad, CA, USA) according to the manufacturer's instructions. Gene expression was quantitatively analyzed by real-time PCR performed using a LightCycler 480 II Real-time PCR Instrument (Roche, Basel, Switzerland) and QuantiFast SYBR Green PCR Master Mix (Qiagen, Hilden, Germany). Primer sequences are listed in Supplementary Table 1.

### Western blotting

Western blotting was performed as previously described.^2^ Briefly, ten pairs of human samples were homogenized in RIPA buffer (Bio-Rad, Hercules, CA, USA) supplemented with PMSF (Kang Chen, Shanghai, China) and protease and phosphatase inhibitors (Roche Applied Science, Shanghai, China), incubated on ice for 30 min, and centrifuged at 12 000 r.p.m. for 10 min. Supernatants were collected and protein concentration was measured by the BCA protein assay (Thermo Fisher Scientific, IL, USA). Proteins were separated by SDS-PAGE and transferred to PVDF membranes (Millipore, Billerica, MA, USA). After blocking with non-fat milk, the membranes were incubated with antibodies against phospho-p65, Collagen I, COX-2, *α*-SMA, *β*-actin (Abcam, Cambridge, MA, USA), and Collagen III (Proteintech, Wuhan, China), followed by incubation with the appropriate secondary antibodies (Cell Signaling Technology, Danvers, MA, USA). The bands were detected with an imaging system (Image Quant LAS 4000, GE Healthcare Life Sciences, Bucking-hamshire, England). Image J version 1.51a (National Institutes of Health, Bethesda, MD, USA) was used to analyze band density.

### Immunohistochemistry

Immunohistochemistry staining with anti-p65 antibody (Proteintech) was performed according to standard protocols and analyzed under a light microscope.

### Tendon injury model

Animal protocols were approved by the Animal Research Committee of Shanghai Jiao Tong University Affiliated Sixth People's Hospital, and all procedures were performed according to the standard guidelines. Adult Sprague–Dawley (SD) rats (weighting 250–300 g) were randomly assigned to the normal and TI groups (*n*=20 per group), and tendon injury was inflicted for the TI group as previously reported,^4, 31^ whereas no surgery was performed in the normal group. For tendon injury, animals were anesthetized by intraperitoneal injection of pentobarbital sodium (40 mg/kg), the right hind limb was cleaned with iodophor, and an incision was made over the medial aspect of the plantar surface, between the interspace of the two medial digits and the ankle. The superficial flexor tendon was removed, and the deep flexor tendon was transected and repaired with 6-0 prolene suture (Ethicon, Edinburgh, UK).

In another experiment, 60 rats were randomly divided into the SO, NC, and p65-siRNA (p65-si) groups (*n*=20 per group). In the SO group, animals were anesthetized and skin was incised, but no tendon injury inflicted, whereas in the other two groups, the surgery for tendon injury was performed. On the third day after surgery, rats in the NC and p65-si groups were subcutaneously injected with 10 nmol of control and p65-specific siRNA (Ribobio, Guangzhou, China), respectively; injections were repeated every 3 days. Fluorescent imaging was performed using IVIS-Lumina-XRMS (PerkinElmer, Hopkinton, MA, USA) at week 1 and 3 after surgery. Real-time PCR and western blotting analyses of skin fibroblasts were used to compare silencing efficiency of three different p65-siRNAs (Supplementary Figure 2) to select the most efficient siRNA for the *in vivo* experiment. The primer used forreal-time PCR is shown in Supplementary Table 2.

The animals were killed 3 weeks after tendon injury and used for analysis.

Macroscopic evaluation was performed in five rats from each group; the severity of peritendinous adhesions was assessed by three independent observers according to the adhesion grading system.^2^

Hematoxylin–eosin (HE) staining and immunohistochemistry using anti-p65 antibody were performed according to standard protocols^2^ and analyzed by light microscopy; five rats from each group were used. Histological evaluation was done by three independent observers according to the histological scoring system.^2^

The work of flexion and maximal tensile strength in five freshly harvested samples in each group were assessed using a biomechanical analyzer (Instron, Norwood, MA, USA) to evaluate tendon adhesion and healing, respectively, as previously described.^2^

Protein expression in tissue samples was analyzed by western blotting as described above using anti-p65 antibody (Abcam, Cambridge, UK) and other antibodies applied for human samples.

### Cell culture

NIH3T3 fibroblast cell line was purchased from ATCC. Tendon cells were harvested from rat superficial flexor tendons collected from the hind paws as previously reported.^2^ Briefly, the tendons were cut into small pieces, digested with 0.15% collagenase NB4 (SERVA Electrophoresis GmbH, Heidelberg, Germany) for 2 h at 37 °C, and the mixture was filtered through a cell mesh (Corning, New York, NY, USA). After centrifugation at 200 × *g* for 5 min, the supernatant was discarded and the cell pellet was resuspended in Dulbecco's modified Eagle media (DMEM) containing 10% fetal bovine serum (FBS; Gibco, Carlsbad, CA, USA). Cells were cultured at 37°C in 5% CO_2_ atmosphere; the medium was replaced every three days. After reaching 80% confluence, tenocytes were lifted from the culture substratum by trypsinization and re-seeded into dishes.

### SiRNA transfection *in vitro*

Fibroblasts and tenocytes were pre-treated or not with 5 ng/ml of transforming growth factor beta 1 (TGF-*β*1; PeproTech, Rocky Hill, NJ, USA) for 48 h to imitate the *in vivo* microenvironment.^23^ Cells were transfected at 50% confluence with negative control siRNA or p65-specific siRNA using Lipofectamine 2000 (Invitrogen) according to the manufacturer's instructions; the sequences of siRNAs are shown in Supplementary Table 3. Six hours after transfection, the medium was replaced by normal fresh medium, and cells were then used for evaluation. Silencing efficiency of three different siRNAs was evaluated by real-time PCR and western blotting (Supplementary Figure 2). Control cells did not receive any treatment.

Cells were collected 48 h after transfection and analyzed for protein expression by western blotting (as described), mRNA expression by real-time PCR, cell proliferation, and flow cytometry.

### Helenalin treatment

Helenalin has been proved as a selective inhibitor of the RelA/p65 subunit.^24^ Cells were pre-treated or not with TGF-*β*1 as described and then treated or not with 1.5 *μ*M Helenalin for 24 h.

### JSH23 treatment

JSH is another selective inhibitor of p65.^25^ Cells pre-treated or not with TGF-*β*1 were cultured with 30 *μ*M JSH23 for 12 h as previously described.^32^

### Cell proliferation assay

Cells were seeded at the a density of 2 × 10^3^ per well into 96-well plates and transfected with siRNA or treated with Helenalin or JSH23 as described above. Cell proliferation was assessed by incubating them with Cell Counting Kit-8 reagents (Dojindo, Shanghai, China) for 4 h at 37 °C and measuring the absorbance at 450 nm. The experiment was performed in triplicate.

### Flow cytometry

Cells were collected 48 h after transfection, stained using the Annexin V-FITC apoptosis detection kit (BD Pharmingen, San Diego, CA, USA) as previously described,^33^ and analyzed in FACSAria flow cytometer (BD Biosciences, San Diego, CA, USA). Five independent samples were used for apoptosis analysis.

### RT-PCR and western blotting

The mRNA and protein expression of p65, Collagen I, Collage III, COX-2, *α*-SMA were analyzed by RT-PCR and western blot as described previously. Five independent samples were used in RT-PCR analysis. Primer sequences are listed in Supplementary Table 2. The antibodies used are described previously.

### Statistical analysis

The data are presented as the means±S.D. Statistical analysis was performed using paired Student *t*-test and one-way ANOVA for multiple comparisons. Differences were considered statistically significant at *P*<0.05.
