# Supplementary material for: RelA/p65 inhibition prevents tendon adhesion by modulating inflammation, cell proliferation, and apoptosis
Source: Cell Death Dis. 2017 Mar 30;8(3):e2710–. doi: 10.1038/cddis.2017.135 (PMC5386538; doi:10.1038/cddis.2017.135)
Supplement: Supplementary figure Legends and Tables [file cddis2017135x1.docx]

**Supplementary Figure 1.** Confirmation of increased genes expression in NF-κB pathway with real-time PCR. The data are shown as the means ± S.D. Statistical significance was calculated using paired Student t-test for 3 independent samples. *P<0.05, **P<0.01.

**Supplementary Figure 2.** Silencing efficiency comparison three different siRNAs by Real-time PCR and Western blot. (A) Comparison for rats *in vivo* study (B) Comparison for mice *in vitro* study.

**Supplementary Tables:**

**Supplementary Table 1. The RT-PCR primer sequences for human**

| **Genes** | **Primer sequences** | |
| --- | --- | --- |
| **NFKBIA** | **Forward** | **AACCTTCAGATGCTGCCA** |
|  | **Reverse** | **AAACACACAGTCATCATAGGG** |
| **IL-6** | **Forward** | **GTTGTTGTTAATGGGCATTCC** |
|  | **Reverse** | **GTGTCCTAACGCTCATACTTT** |
| **GADD45B** | **Forward** | **GGAGTGAGACTGACTGCAA** |
|  | **Reverse** | **TCCCAAGTCCCAAAGTGTG** |
| **CXCL2** | **Forward** | **CAGAAAGCTTGTCTCAACCC** |
|  | **Reverse** | **TCAGGAACAGCCACCAAT** |
| **RELB** | **Forward** | **GGAAGTAGACATGAATGTGGTG** |
|  | **Reverse** | **CGCAGCTCTGATGTGTTT** |
| **MYD88** | **Forward** | **TCTGAGCCATTCACACATCT** |
|  | **Reverse** | **TTCACCATTTCCTACAGGGATT** |
| **ACTB** | **Forward** | **CCATCATGAAGTGTGACG** |
|  | **Reverse** | **GCCGATCCACACGGAGTA** |

**Supplementary Table 2. The RT-PCR primer sequences for mouse and rat**

| **Genes** | **Primer sequences** | |
| --- | --- | --- |
| **Rela (rat)** | **Forward** | **CTGGCCATGGACGATCTGTT** |
|  | **Reverse** | **GCACTTGTAACGGAAACGCAT** |
| **Rela** **(mouse)** | **Forw****ard** | **TCCTGTTCGAGTCTCCATGCAG** |
|  | **Reverse** | **GGTCTCATAGGTCCTTTTGCGC** |
| **COL1 (Collagen I, mouse)** | **Forward** | **CCTCAGGGTATTGCTGGACAAC** |
|  | **Reverse** | **CAGAAGGACCTTGTTTGCCAGG** |
| **COL1 (Collagen III (mouse)** | **Forward** | **AGCCACCTTGGTCAGTCCTA** |
|  | **Reverse** | **GTGTAGAAGGCTGTGGGCAT** |
| **Acta2 (α-SMA, mouse)** | **Forward** | **ACCATGAAGATCAAGATCATTGC** |
|  | **Reverse** | **TGTGTGCTAGAGGCAGAGC** |
| **COX2 (COX-2, mouse)** | **Forward** | **ACCGAGTCGTTCTGCCAATA** |
|  | **Reverse** | **TAAGTCCTAGTGAGGGGACGG** |

**Supplementary Table 3. The siRNA sequences for rat and mouse.**

| **Genes** | **SiRNA sequences** | | |
| --- | --- | --- | --- |
| **Rela (rat)** | **SiRNA-1** | **Sense** | **GTGGCCATTGTGTTCCGAA dTdT** |
|  |  | **Antisense** | **TTCGGAACACAATGGCCAC dTdT** |
|  | **SiRNA-2** | **Sense** | **GGACCTACGAGACCTTCAA dTdT** |
|  |  | **Antisense** | **TTGAAGGTCTCGTAGGTCC dTdT** |
|  | **SiRNA-3** | **Sense** | **GAGCATCATGAAGAAGAGT dTdT** |
|  |  | **Antisense** | **ACTCTTCTTCATGATGCTC dTdT** |
| **Rela (mouse)** | **SiRNA-1** | **Sense** | **GACATTGAGGTGTATTTCA dTdT** |
|  |  | **Antisense** | **TGAAATACACCTCAATGTC dTdT** |
|  | **SiRNA-2** | **Sense** | **GCGAAUCCAGACCAACAAU dTdT** |
|  |  | **Antisense** | **AUUGUUGGUCUGGAUUCGC dTdT** |
|  | **SiRNA-3** | **Sense** | **GGACCUAUGAGACCUUCAA dTdT** |
|  |  | **Antisense** | **UUGAAGGUCUCAUAGGUCC dTdT** |
